# Supplementary material for: Ablation of GPR56 Causes β-Cell Dysfunction by ATP Loss through Mistargeting of Mitochondrial VDAC1 to the Plasma Membrane
Source: Biomolecules. 2023 Mar 18;13(3):557. doi: 10.3390/biom13030557 (PMC10046417; doi:10.3390/biom13030557)
Supplement: Supplementary file 1 [file biomolecules-13-00557-s001.zip › biomolecules-2210727-supplementary.pdf]

Supplementary Tables

Table S1. The list and identities of siRNA and shRNA used in the study.

GPR56 shRNA (m) Lentiviral Particles	Santa Cruz	sc-60750-V
GPR56 (ADGRG1) siRNA (r)	ThermoFisher Scientific	s141373
GPR56 (ADGRG1) siRNA (r)	ThermoFisher Scientific	s141374
GPR56 (ADGRG1) siRNA (r)	ThermoFisher Scientific	s141375
GPR56 (ADGRG1) siRNA (h)	ThermoFisher Scientific	s17757
GPR56 (ADGRG1) siRNA (h)	ThermoFisher Scientific	s17759
GPR56 (ADGRG1) siRNA (h)	ThermoFisher Scientific	s17758

Table S2. Primer sequence identities used in the study.

VDAC1 primer (rat)	AB applied biosystem	Rn00821325_g1
VDAC2 primer (rat)	AB applied biosystem	Rn00755819_m1
ChREBP primer (rat)	AB applied biosystem	Rn00591943_m1
Txnip primer (rat)	AB applied biosystem	Rn01533891_g1
HPRT primer (rat)	AB applied biosystem	Rn01527840_m1
PPIA primer (rat)	AB applied biosystem	Rn00690933_m1
VDAC1 primer (human)	AB applied biosystem	Hs01631624_gH
HPRT primer (human)	ThermoFisher Scientific	Hs01003267_m1
PPIA primer (human)	AB applied biosystem	Hs04194521_s1

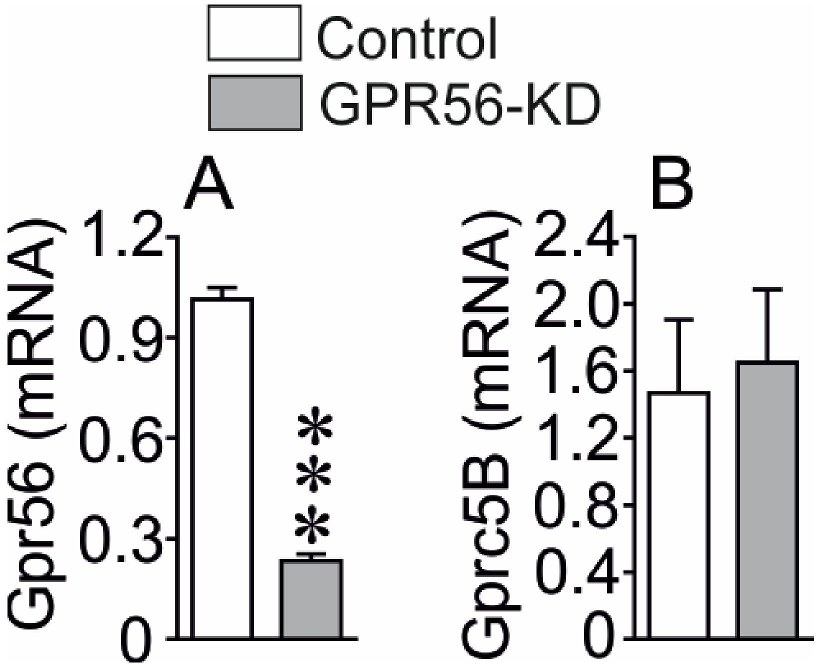

Figure S1. The efficiency of *GPR56*-KD in INS-1 832/13 cells. Analysis of Gpr56 mRNA by qPCR showing a reduced Gpr56 expression in Gpr56-KD (internal control of KD efficiency) (A) and unchanged expression of Gprc5B (off-target effect control) (B) compared to scramble controls INS-1 832/13 cells. Mean±SEM from four different experiments in each group are shown. ***p <0.001.

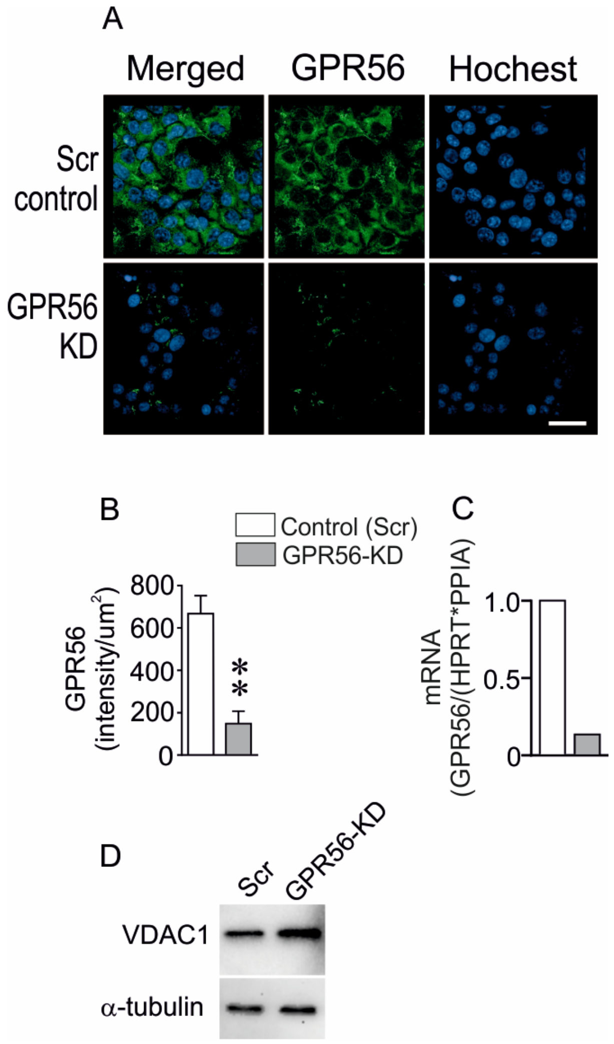

Figure S2. The impact of GPR56-KD on GPR56 and VDAC1 protein expression in EndoC βH1 cells. The detection of protein measured by confocal microscopy (A and B) and detection of mRNA analysis by qPCR (C) shows a marked reduction in the GPR56 expression after GPR56-KD in EndoC βH1 cells. A representative confocal microscopic image (A) and the cellular GPR56 expression intensity (B) are shown. Mean±SEM for n=3 different experiments are shown. Analysis of GPR56 mRNA in an experiment for scramble control and GPR56-KD cells. Hoechst detection by confocal microscopy in the same cells is also illustrated (A). VDAC1 protein is increased in GPR56-KD EndoC βH1 cells and the results are from four different experiments (D). P<0.01. Bar indicates 20 um.

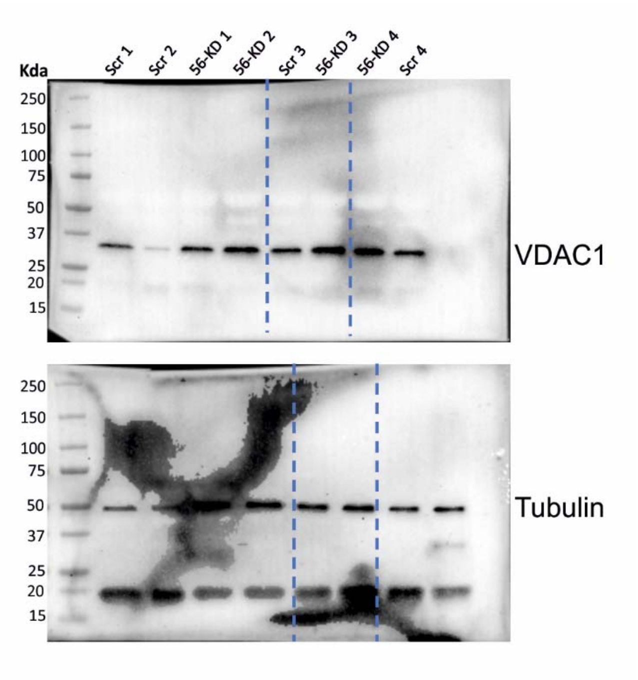

Figure S3 (related to Figure S2D). Original Western blot images showing VDAC1 and Tubulin protein expression in scramble control (Scr) and GPR56-KD (56-KD) EndoC βH1 cells from four different experiments. The dotted lines denote the bands shown in Figure S2D.
